# Antiresistin RNA Oligonucleotide Ameliorates Diet-Induced Nonalcoholic Fatty Liver Disease in Mice through Attenuating Proinflammatory Cytokines

**DOI:** 10.1155/2015/414860

**Published:** 2015-04-01

**Authors:** Yi Tan, Xing Liang Jin, Weiguo Lao, Jane Kim, Linda Xiao, Xianqin Qu

**Affiliations:** School of Medical & Molecular Biosciences, University of Technology Sydney, Sydney, NSW 2007, Australia

## Abstract

The aim of this study was to determine whether inhibition of resistin by a synthetic antiresistin RNA (oligonucleotide) oligo ameliorates metabolic and histological abnormalities in nonalcoholic fatty liver disease (NAFLD) induced by high-fat diet (HFD) in mice. The antiresistin RNA oligo and a scrambled control oligo (25 mg/kg of body weight) were i.p. injected to HFD mice. Serum metabolic parameters and hepatic enzymes were measured after 4-week treatment. The treatment significantly reduced epididymal fat and attenuated the elevated serum resistin, cholesterol, triglycerides, glucose, and insulin with an improved glucose tolerance test. Antiresistin RNA oligo also normalized serum AST and ALT levels with improved pathohistology of NAFLD. Immunoblotting and qRT-PCR revealed that decreased protein and mRNA expression of resistin in fat and liver tissues of the treated mice were associated with reduction of adipose TNF-*α* and IL-6 expression and secretion into circulation. mRNA and protein expression of hepatic phosphoenolpyruvate carboxykinase (PEPCK) and sterol regulatory element-binding protein-1c (SREBP-1c) were also significantly decreased in the treated mice. Our results suggest that resistin may exacerbate NAFLD in metabolic syndrome through upregulating inflammatory cytokines and hepatic PEPCK and SREBP-1c. Antiresistin RNA oligo ameliorated metabolic abnormalities and histopathology of NAFLD through attenuating proinflammatory cytokines.

## 1. Introduction

Nonalcoholic fatty liver disease (NAFLD) is emerging as an important public health problem worldwide. NAFLD is classified from the range of simple steatosis and nonalcoholic steatohepatitis (NASH) followed by fibrosis [1]. The pathology of NAFLD is characterized by excessive deposition of free fatty acids (FFAs) and triglycerides (TG) in the hepatic parenchyma [2]. NAFLD is a multifactorial disease coupled with clinical hallmarks of metabolic syndrome (MetS) including obesity, insulin resistance, dyslipidemia, and lower grade inflammation [3]. Recently, increased prevalence of NAFLD has been reported in patients with MetS and polycystic ovary syndrome (PCOS) [4, 5], suggesting that the development of NAFLD, PCOS, and MetS shares some common pathogenesis, for example, central obesity with increased adipocyte-derived cytokines and inflammatory processes [6, 7]. Increased evidence indicates that excessive abdominal fat associated with ectopic fat deposition in nonadipose tissues exacerbates inflammation and lipotoxicity through releasing various adipose-derived proteins, termed “adipokines,” into the circulation, leading to MetS and NAFLD [3, 8].

Of the identified adipose-derived adipokines, resistin seems to assert its effects on both inflammatory and insulin signalling pathways [9]. Resistin was originally discovered in the adipose tissue of mice and named for its ability to resist (interfere with) insulin action [10]. Animal studies have highlighted the ability of resistin to induce skeletal muscle and hepatic insulin resistance after both acute and chronic administration [11, 12]. Increasing evidence from clinical studies suggests that resistin is implicated in various human pathologies, including MetS, type 2 diabetes, cardiovascular disease [CVD], and obesity-related subclinical inflammation [13–16], but the role of resistin in the development of NAFLD is controversial. A few clinical studies have reported that serum resistin levels did not differ between patients with NAFLD and without the disease [17]. However, one study has shown that excessive ectopic accumulation of fat in the liver and skeletal muscle of insulin-resistant subjects is associated with lower concentrations of serum resistin [18], while another reports increased levels of circulating resistin, but only in patients with severe liver disease [19]. These inconsistent findings indicate that the role of resistin in NAFLD should be further clarified.

The pathogenesis of NAFLD is positively correlated to overnutrition or inappropriate diet which leads to chronic elevated circulating glucose, insulin, and FFA [20]. Our previous studies have demonstrated that high-fat diet (HFD) induces metabolic syndrome accompanied by an increased accumulation of TG in the liver of rats and mice [21, 22]. Another study has shown that HFD-induced obesity in rodents is associated with the elevation of serum resistin levels and hepatic insulin resistance [23]. However, it remains to be elucidated as to whether increased resistin expression and secretion are directly implicated in NAFLD. Unlike murine resistin, resistin is mainly secreted by macrophages in humans [24], suggesting that resistin is linked to inflammation which is crucial in the pathogenesis of NAFLD [25]. The illustration of this relationship between resistin expression and elevated proinflammatory cytokines, such as tumor necrosis factor-*α* (TNF-*α*) and interleukins (ILs), will help to understand the pathogenesis of NAFLD. Furthermore, whether overexpression of resistin is involved in abnormal hepatic* de novo* lipogenesis remains unclear. In the present study, a synthetic RNA oligonucleotide (oligo) was designed to target the mouse resistin gene (antiresistin RNA oligo) as a pharmacological tool to clarify the actual role of resistin in HFD-induced NAFLD in mice. Furthermore, whether inhibition of resistin with antiresistin RNA oligo ameliorates HFD-induced metabolic syndrome and NAFLD and the possible molecular mechanisms were investigated.

## 2. Material and Methods

### 2.1. Design of RNA Oligonucleotide (Oligo) against Resistin mRNA

RNA oligo was designed to complement the start codon region of the mouse resistin gene (GenBank: AF323080.1) along the resistin mRNA based on the free energy analysis. The RNA oligo was modified with 2′-O-methyl and a 3′-butanol cap to increase the* in vivo* stability of RNA. The sequences of RNA oligo against resistin (antiresistin oligo) and the scrambled control RNA oligo (control oligo) were 5′-GGG AAA UGA AAG GUU CUU CAU-3′ and 5′-AGA CCU CUC AUA GCA GCU GAT-3′, respectively. To validate synthetic RNA oligo could effectively block resistin expression, antiresistin oligo and the control oligo were transfected into 3T3-L1 adipocytes. The efficiency of synthetic RNA oligo to block resistin expression in transfected 3T3-L1 cell was 97% determined by quantitative real-time polymerase chain reactions (qRT-PCR) assay as described previously [22].

### 2.2. Animals and Treatment

Male C57BL/6 mice (8 weeks of age with average body weight 20.87 g) supplied by the Animal Resources Center (Perth, Australia) were acclimatized in communal cages at 22°C, with a 12 h light, 12 h dark cycle (lights on at 0700) for 1 week and had access to a standard chow or a HFD (59% fat, 21% protein, and 20% carbohydrate by energy composition) for 10 weeks to induce insulin resistance and fatty liver disease. Body weight and food intake were measured weekly. Antiresistin RNA oligo and control oligo were freshly prepared in medical saline at the ratio of 25 mg per 5 mL. The treatment dosage and its duration were designed to ensure the maximal stable inhibition of the gene transcript of interest, which was derived from our previous study [22]. After 10-week HFD feeding, HFD mice were randomly divided into two groups (*n* = 10) and treated with scrambled control oligo (25 mg/kg of body weight) or antiresistin oligo (25 mg/kg of body weight) via i.p. injection every second day for 4 weeks. HFD feeding was continued during 4 weeks of treatment. 10 chow fed mice were treated with saline (5 mL/kg of body weight) i.p. injection as a normal control group to HFD fed mice with the control oligo treatment.

All experimental procedures were approved by the Joint Royal North Shore Hospital/University of Technology Sydney Animal Care and Ethics Committee and were in accordance with the National Health and Medical Research Council of Australia Guidelines on Animal Experimentation.

### 2.3. Intraperitoneal Glucose Tolerance Tests

At the end of 4 weeks' treatment, all mice were subjected to the intraperitoneal glucose tolerance test (i.p.GTT) following an overnight fasting (12 hours). A glucose bolus (2 g/kg of body weight) was i.p. injected to the conscious, unrestrained mice. The blood glucose levels were determined from tail blood samples taken at 0 (prior to glucose administration), 15, 30, 60, 90, 120, and 150 min after the glucose injection using One Touch Profile glucometer. Results of i.p.GTT were expressed as integrated areas under the curves (AUC) over 150 min for glucose calculated using the Trapezoidal Rule with Graphpad prism 4 software (Graphpad Prism, CA, USA).

### 2.4. Blood and Tissue Sample Collection

At the end of the experiment, mice were fasted overnight (12 hours) and then deeply anaesthetized with inhalant aesthetic gas (isoflurane and nitrous oxide). Blood samples were collected from the heart and centrifuged (10 min at 1400 g) to separate the serum fraction for biochemistry assays. Liver and visceral adipose tissue were quickly excised and then stored at −80°C for later measurements.

### 2.5. Measurement of Metabolic Parameters

Fasting serum total cholesterols, TG, and nonesterified fatty acids (NEFAs) were analysed using enzymatic colorimetric kits obtained from Roche Diagnostics GmbH (Mannheim, Germany) and Wako Pure Chemical Industries (Osaka, Japan), respectively. Fasting serum insulin concentration was measured using ELISA kits (Linco Diagnostic Services, MO, USA). Fasting serum glucose, ALT, and AST were determined by spectrophotometric analysis using commercial kits (Dialab, Vienna, Austria). Hepatic TG was determined from liver tissue extraction using the chloroform/methanol method (2 : 1) described previously [16]. Whole-body insulin sensitivity was estimated according to the homeostasis model assessment of insulin resistance (HOMA-IR) using the formula: [fasting serum glucose (mmol) times fasting serum insulin (mU/mL)]/22.5.

### 2.6. Liver Histological Analysis

A small portion of frozen liver tissue (*n* = 10) was cut and embedded with precooled optimal cutting compound (Torrance, CA, USA) for cryostat sectioning at 6 *μ*m. The sections were mounted on microscope slides then fixed with 10% formaldehyde solution. The samples were then stained with Haematoxylin and Eosin (H&E) or Oil Red O (Sigma-Aldrich, St. Louis, MO, USA). H&E-stained slides were observed under light microscopy (Olympus, BX51 microscope, Tokyo, Japan) to investigate the architecture of liver and hepatocyte steatosis. Images were captured using an Olympus digital camera (DP70, Tokyo, Japan) with a sample size at least 80 fields per group (original magnification, ×400, 36-bit colour, 1280 × 1024 pixels). Liver steatosis was graded with semiquantitative estimation of the percentage of lipid-laden hepatocytes, according to the method previously described [18]. At least 5 different high-power fields (original magnification, ×400) were graded in a blinded way. Stained Oil Red O slides were visualized with the Olympus microscope and images were captured with digital camera (DP70, Tokyo, Japan) using Image-Pro 6.2 software (Media Cybernetics, Inc., MD, USA).

### 2.7. Measurement of Resistin and Proinflammatory Cytokines in Circulation

Serum resistin concentrations were measured by a commercial ELISA kit (BD Biosciences, NSW, Australia) according to the manufacturer's instructions. Serum analysis of adiponectin was based on a commercially available mouse adiponectin ELISA kit (EZMADP-60K; Millipore, MO, USA). Serum levels of TNF-*α*, interleukins (IL-6 and IL-10), interferon gamma (IFN*γ*), and leptin were measured with a standard sandwich enzyme immunoassay method using the Bio-Plex Pro mouse cytokine assay kit (Bio-Rad Laboratories, Hercules, CA, USA) according to the manufacturer's instructions. Resulting levels of TNF-*α*, ILs, IFN*γ*, leptin, and adiponectin were determined using a Bio-Plex MAGPIX array reader, which identified and quantified each specific reaction based on bead colour and fluorescent signal intensity and expressed as pictogram per millilitre.

### 2.8. Quantitative RT-PCR Assay of mRNA Expressions in Adipose Tissue and Liver

The expression of mRNAs for resistin, the proinflammatory cytokines (TNF-*α* and IL-6), leptin, adiponectin in adipose tissue, TNF-*α*, IL-6, PEPCK, and SREBP-1c in the liver were determined by quantitative real-time polymerase chain reactions (qRT-PCR). Total RNA was extracted from 100 mg of frozen visceral fat or liver tissue of mice using TRIzol reagent (Invitrogen, Mulgrave, VIC, Australia) according to the manufacturer's protocol. After determination of RNA concentrations by measuring the absorbance at 260/280 nm 0.5 *μ*g RNA as template was reversely transcribed to cDNA by using QuantiTect Reverse Transcription kit (QIANGEN, Alabama, USA). cDNA was amplified using PCR assay with the mouse primer sequences listed in Table 1 (Sigma, CT, USA).

*β*-actin was used as reference genes from Promega (Madison, WI, USA). The amplification was performed with fastStart Universal SYBR Green Master (Roche Diagnostics, Mannheim, Germany) in Mastercycler ep realplex (Eppendorf, CA, USA). The relative mRNA levels in antiresistin oligo treated mice were presented as a percentage of mRNA levels in control oligo group.

### 2.9. Immunoblotting Assays

Expressions of resistin protein in adipose and liver tissues as well as hepatic PEPCK and SREBP-1c were detected by immunoblot analysis. 100 mg of frozen liver or visceral fat samples was homogenized in 1 mL lysis buffer (Roche Diagnostics, Indianapolis, IN, USA). Then, the homogenates were centrifuged at 3000 ×g for 10 min at 4°C. The supernatants were collected and the protein concentration of each supernatant was determined by the Bradford method. Total protein (20 *μ*g) was separated to SDS-PAGE (4–20%) and electroblotted onto PVDF membranes. Blotted membranes were blocked with 5% skim milk in PBS with 0.05% Tween 20 and incubated with monoclonal antiresistin diluted at 1 : 3000 (ALEXIS Biochemicals, San Diego, CA, USA), anti-PEPCK diluted at 1 : 1000 (Agrisera, Vannas, Sweden), anti-SREBP-1c, and anti-*β*-actin diluted at 1 : 1000 (SC-367; Santa Cruz Biotechnology, CA, USA), respectively, and incubated overnight at 4°C; then the corresponding horseradish peroxidase-conjugated secondary antibody (1 : 10,000) was incubated for 2 h. Protein expressions were visualized by the ECL system (Perce, Rockford, IL) and analyzed using Quantity One 4.6.1 software of the ChemiDoc XRS system (Bio-Rad Laboratories, Hercules, CA, USA).

### 2.10. Statistical Analyses

All values are expressed as mean ± SE. Comparisons across the three groups were performed using one-way analysis of variance (ANOVA) followed by Tukey test to determine significant differences between two groups using Prism version 5 (GraphPad Inc., San Diego, CA). Coefficient of correlation was performed to evaluate whether there is any correlation between serum levels of resistin and changes of HOMA-IR and proinflammatory cytokines (TNF-*α* and IL-6). *P* value <0.05 was considered statistically significant.

## 3. Results

### 3.1. Decreased Resistin Expression and Secretion by Antiresistin RNA Oligonucleotide

**Figure 1:**
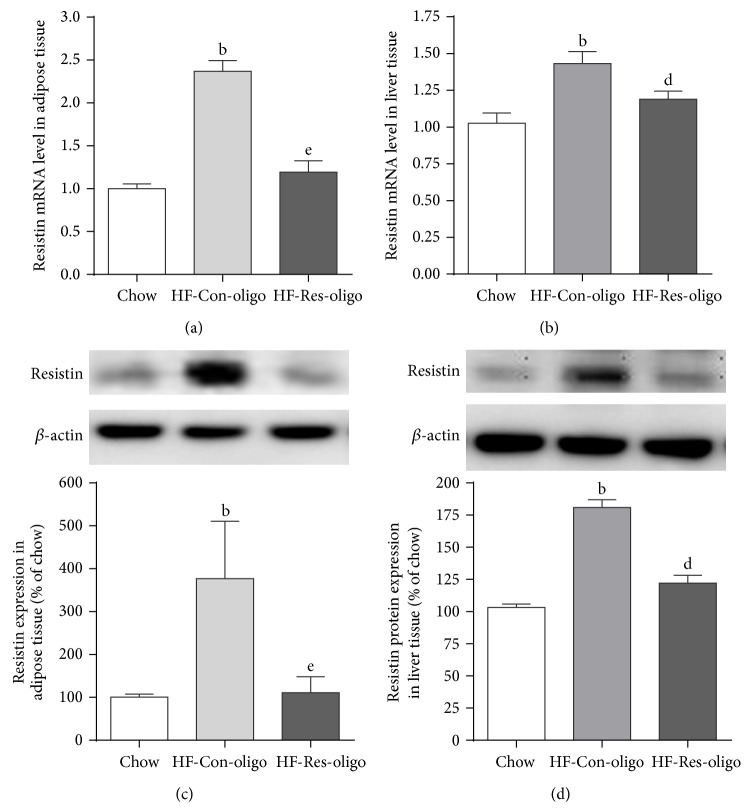
Effects of synthetic RNA oligo on resistin expression and secretion. Immunoblots and quantitative analysis of resistin protein in adipose tissue (a) and in the liver (b); relative mRNA expression was quantified by qRT-PCR in adipose tissue (c) and in the liver (d). Data are mean ± SE from 10 mice each group. ^b^ *P* < 0.01 versus chow fed mice; ^e^ *P* < 0.01 versus HF-Con-oligo mice.

By a total of 14 weeks of HFD exposure, C57BL/6 mice increased epididymal fat mass and developed metabolic syndrome with the elevated serum AST and ALT levels when compared with chow fed mice (Table 2). In association with these metabolic changes, ELISA analyses revealed that serum resistin levels were significantly higher in HFD control mice than chow fed mice (Table 2, *P* < 0.01). Expression of resistin mRNA was increased by 139.6% in adipose tissue and 46.6% in the liver of HFD mice compared with chow fed mice (Figures 1(a) and 1(b), *P* < 0.01 and 0.01, resp.). qRT-PCR analysis also revealed that the increased expression of resistin mRNA was markedly blocked by antiresistin RNA oligo in adipose tissue and liver (Figures 1(a) and 1(b), *P* < 0.01 and *P* < 0.05; HF-Res-oligo group versus HF-Con-oligo group). HFD increased resistin protein expression by 190.3% in adipose tissue and 73.3% in the liver (Figures 1(c) and 1(d), HFD-control oligo mice versus chow group, *P* < 0.01) and the 4 weeks' treatment with antiresistin oligo prevented overexpression of resistin protein in adipose and liver tissues (Figures 1(c) and 1(d), *P* < 0.01).

These results indicate that the synthetic RNA oligo effectively blocked resistin gene expression and reduced its translation into protein, which resulted in a decreased secretion of resistin into the circulation (Table 2, *P* < 0.01) in antiresistin oligo treated mice.

### 3.2. Effects of Antiresistin RNA Oligo on Body Weight, Metabolic Parameters, and Hepatic Enzymes

Table 2 shows that the body weight of HFD fed mice was significantly higher than chow fed mice (*P* < 0.05). The body weight gain was slightly lower in antiresistin treated group than the control oligo group but there was no statistical significance. However, the weight of epididymal fat mass was significantly lower in antiresistin oligo treated mice when compared with the Con-oligo group (*P* < 0.05). HFD feeding significantly increased fasting serum glucose level (*P* < 0.05 HFD versus chow fed mice) associated with 30.7% elevation of serum insulin levels (*P* < 0.05), indicating insulin resistance. Antiresistin oligo treatment significantly decreased fasting serum glucose (Table 2, Res-oligo versus C-oligo group, *P* < 0.05) associated with improved insulin resistance, evidenced by a reduction of fasting serum insulin levels (*P* < 0.01) and reduced HOMA-IR values (Table 2, *P* < 0.05). Antiresistin oligo also significantly reduced the elevated serum levels of cholesterol, TG, and NEFAs levels by 27.9%, 20.1%, and 19.4%, respectively, (all *P* < 0.05) when compared with the HF-Con oligo group. Serum hepatic enzyme (AST and ALT) levels in HFD fed mice were increased by 3.0- and 6.2-fold, respectively, when compared with chow fed mice (Table 2, both 0.001). Antiresistin oligo treatment significantly attenuated the elevated AST and ALT values (*P* < 0.05 and *P* < 0.001, resp.) when compared with control oligo treated mice (Table 2).

### 3.3. Effect of Antiresistin RNA Oligo on i.p.GTT

**Figure 2:**
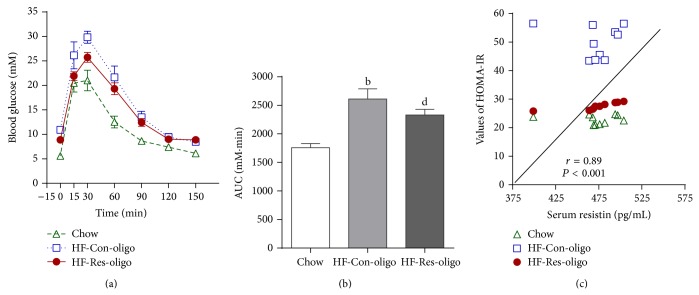
Effects of antiresistin RNA oligo on intraperitoneal i.p. glucose tolerance test. (a) Blood glucose response to i.p. glucose injection, (b) area under the curves (AUC) of glucose profile values in chow fed mice and HFD fed mice treated with a control oligo or antiresistin RNA oligo, and (c) the correlations between serum levels of resistin and whole-body insulin sensitivity index (HOMA-IR). Data are mean ± SE (*n* = 10 each group). ^b^ *P* < 0.01 versus chow fed mice; ^e^ *P* < 0.05 versus HF-Con-oligo mice.

Impaired glucose tolerance was induced by HFD feeding, showing significantly increased AUC of glucose profile values by 32.7% over the entire i.p.GTT (Figures 2(a) and 2(b), AUG 1757 ± 61 versus 2610 ± 179 mM·min, *P* < 0.01). Blood glucose disposal was significantly faster in antiresistin RNA oligo treated mice, evidenced by 29.7% reduction of AUC values compared with the HF-control oligo group (Figure 2, *P* < 0.05). The results of i.p.GTT together with HOMA-IR (Table 2) indicate that antiresistin RNA oligo improved whole-body insulin resistance in HFD mice. Reduction of serum levels of resistin was positively correlated to the enhanced insulin sensitivity in antiresistin RNA oligo treated mice (Figure 2(c), *P* < 0.001).

### 3.4. Effect of Antiresistin RNA Oligo on Lipid Deposition in the Liver and Hepatic Steatosis

**Figure 3:**
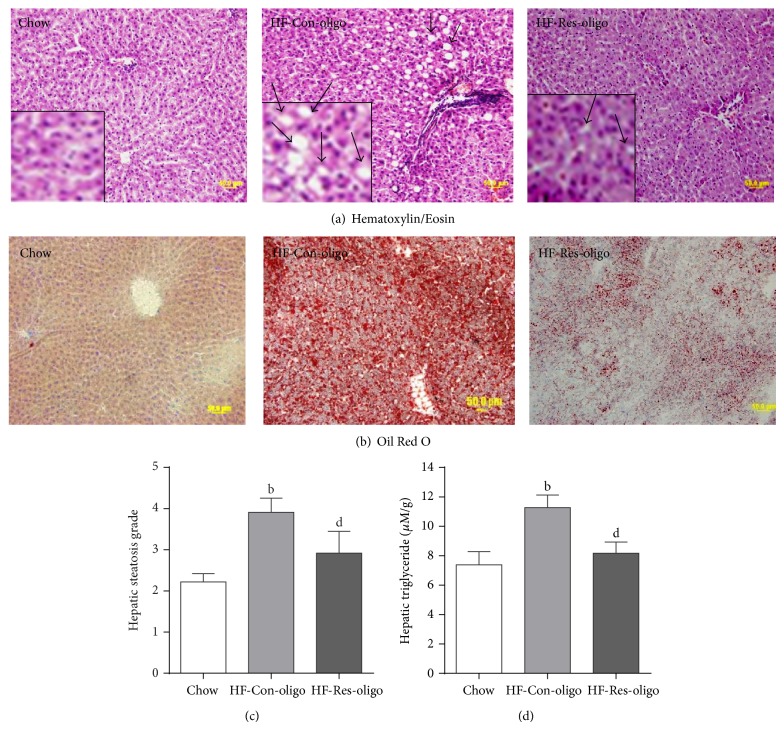
Effects of antiresistin RNA oligo on lipid deposition in the liver and hepatic steatosis. Sections of liver from chow fed mice and HFD fed mice treated with a control oligo or antiresistin oligo were stained with Hematoxylin/Eosin (a). Bottom left corner shows enlarged views of inset image. The arrows show hepatic steatosis and Oil Red O (b) to visualize lipid droplets. The hepatic steatosis grade was based on the percentage of steatotic hepatocytes in the H&E-stained liver sections (c). Hepatic tissue triglyceride levels (d) in all groups of mice. Data are mean ± SE. *n* = 10. ^b^ *P* < 0.01 versus chow fed mice; ^d^ *P* < 0.05 versus HF-Con-oligo mice.

The photomicrographs of the HE stain showed that the majority of the hepatocytes of HFD mice were distended by fat accumulation in comparison to the chow group (Figure 3(a)). Analysis of blindly scored HE-stained sections showed a statistically significant increase in lipid-laden hepatocytes in the liver tissue of control oligo treated mice (Figure 3(c), *P* < 0.05 versus chow fed mice). The treatment with antiresistin oligo decreased the grade of hepatic steatosis compared with control oligo group (Figures 3(a) and 3(c)), indicating that antiresistin RNA oligo greatly prevented lipid infiltrations and hepatic steatosis. Oil Red O staining exhibited many lipid droplets in the liver sections of HFD control mice (Figure 3(b)), whereas few or no lipid droplets were seen in the liver sections from the chow and antiresistin RNA oligo treated HFD mice (Figure 3(b)). Consistent with histological appearance, the hepatic TG levels were significantly higher in HFD fed mice than those in chow fed group (Figure 3(d), 11.3 ± 0.9 versus 7.4 ± 0.9 *μ*M/g, *P* < 0.05). This increase was significantly prevented by antiresistin RNA oligo treatment (Figure 3(d), *P* < 0.05 versus Con-oligo group). Together with normalized serum AST and ALT levels (Table 2), these findings indicate that inhibitions of resistin expression and secretion with RNA oligo can improve HFD-induced pathological changes in NAFLD.

### 3.5. Effects of Antiresistin RNA Oligo on Serum Proinflammatory Cytokines, Leptin, and Adiponectin

**Figure 4:**
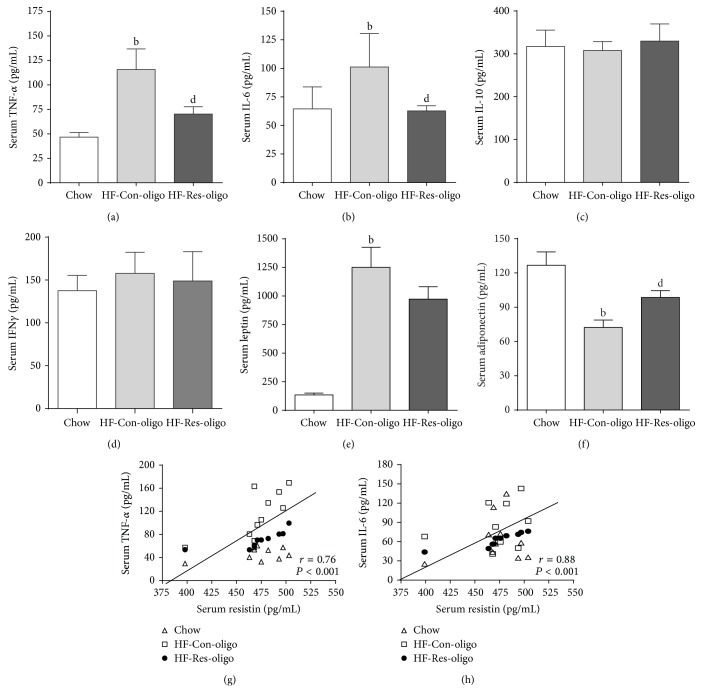
Effects of antiresistin RNA oligo on circulating proinflammatory cytokines, leptin, and adiponectin. Serum TNF-*α*, IL-6, IL-10, and IFN*γ* (a–d) and leptin and adiponectin levels (e-f) were determined in chow fed mice and HFD fed mice treated with a control oligo or antiresistin oligo. The correlations between the differences of serum resistin and both the differences of serum TNF-*α* (g) and IL-6 (h). Data are mean ± SE (*n* = 10). ^a^ *P* < 0.05 and ^b^ *P* < 0.01 versus chow fed mice; ^d^ *P* < 0.05 versus HFD-Con-oligo mice.

Figure 4 shows significantly increased serum TNF-*α*, IL-6, and leptin associated with 55.6% reduction of adiponectin in HF-control mice as compared with the chow group (*P* < 0.05), whereas antiresistin oligo treatment markedly decreased the elevation of TNF-*α* and IL-6 (Figures 4(a) and 4(b), both *P* < 0.05). There were neither significant alterations of circulating IL-10 and IFN*γ* levels by HFD feeding nor changes of serum leptin level by antiresistin oligo treatment (Figures 4(c), 4(d), and 4(e)). Interestingly, the levels of serum adiponectin were the lowest in the HF-control group and increased by 36.3% in antiresistin oligo treated group (Figure 4(f), *P* < 0.05 versus HF-control).

According to the fact that resistin is mainly secreted by macrophages in humans we hypothesised that resistin implicates NAFLD through upregulating secretion of proinflammatory cytokines from adipose tissue. The illustration of the relationship between circulating levels of resistin and proinflammatory cytokines will help to understand underlying mechanism by which antiresistin RNA oligo improved NAFLD pathology. Figures 4(g) and 4(h) showed reduced serum resistin was correlatively associated with decreased serum levels of TNF-*α* and IL-6 (both *P* < 0.001).

### 3.6. Effects of Antiresistin RNA Oligo on mRNA Expressions of Proinflammatory Cytokines, PEPCK and SREBP-1c

**Figure 5:**
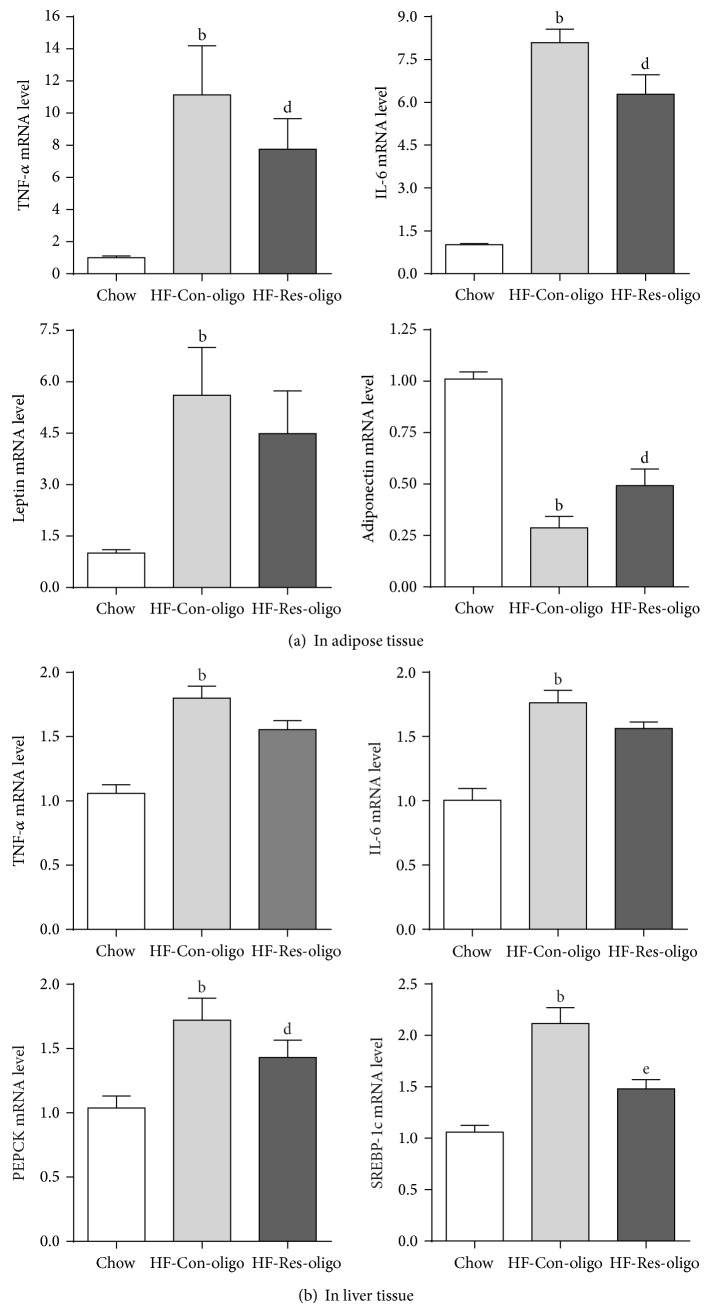
Effects of antiresistin RNA oligo on mRNA expression. (a) TNF-*α*, IL-6, leptin, and adiponectin mRNA expression in adipose tissue; (b) hepatic mRNA expression of TNF-*α*, IL-6, PEPCK, and SREBP-1c in chow fed mice and HFD fed mice treated with a control oligo or antiresistin oligo. Data are mean ± SE (*n* = 10). ^a^ *P* < 0.05 and ^b^ *P* < 0.01 versus chow fed mice; ^d^ *P* < 0.05 versus HFD-Con-oligo mice.

Figure 5(a) shows that mRNA expressions of TNF-*α*, IL-6, and leptin in adipose tissue were significantly higher in the HFD control mice as compared with chow fed mice. These results were consistent with the measurements in the circulation, indicating that HFD feeding induced TNF-*α*, IL-6, and leptin gene expression and secretion. Inhibition of resistin with RNA oligo was associated with reduced mRNA expressions of TNF-*α*, IL-6, and increased adiponectin (Figure 5(a), all *P* < 0.05) but antiresistin oligo did not affect leptin gene expression (Figure 5(a)). In the liver, TNF-*α*, IL-6, and mRNA also significantly increased by HFD feeding and decreased by antiresistin oligo treatment (Figure 5(b)). To clarify whether resistin implicates hepatic glucose production and hepatic fatty acid synthesis, the gene expressions involved in hepatic gluconeogenesis and lipogenesis pathways were detected. mRNA expression of PEPCK and SREBP-1c was significantly increased in HFD control group as compared with chow fed mice. The mRNA of PEPCK in antiresistin oligo treated mice was significantly lower than HF-control group (Figure 5(b), *P* < 0.05). SREBP-1c mRNA was reduced by 68.5% in antiresistin oligo treated mice compared with the HFD control (*P* < 0.01).

### 3.7. Effects of Antiresistin RNA Oligo on Expressions of PEPCK and SREBP-1c in the Liver

**Figure 6:**
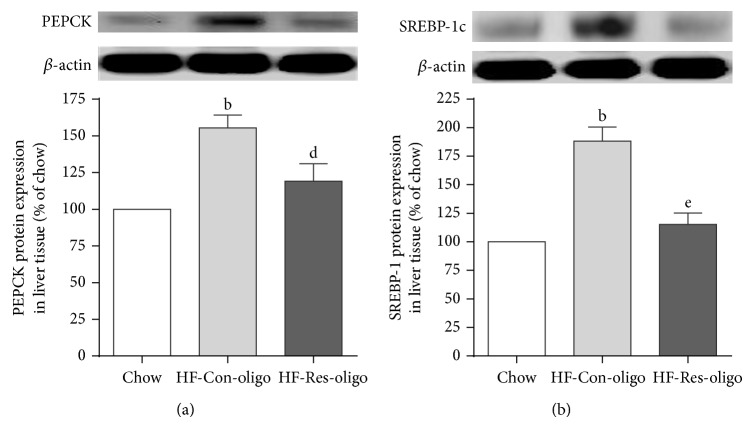
Effects of antiresistin RNA oligo on PEPCK and SREBP1c expression in the liver. Immunoblots and quantitative analysis of PEPCK (a) and SREBP-1c (b) protein in liver tissue of chow fed mice and HFD fed mice treated with a control oligo or antiresistin oligo. Data are mean ± SE (*n* = 10). ^b^ *P* < 0.01 versus chow fed mice; ^d^ *P* < 0.05 and ^e^ *P* < 0.01 versus HFD-Con-oligo mice.

To clarify whether inhibition of resistin mRNA alters protein expression of PEPCK and SREBP-1c both molecules in the liver were detected by immunoblotting. When compared with chow group, chronic HFD feeding led to 50% increase in PEPCK protein content in the liver (Figure 6(a), *P* < 0.01). Antiresistin oligo treatment significantly decreased PEPCK protein expression compared to control oligo treatment (Figure 6(a), *P* < 0.05). HFD feeding also led to 75% increase in SREBP-1c protein expression in the liver (Figure 6(b), *P* < 0.01). Antiresistin oligo treatment reversed overexpression of SREBP-1c protein compared to control oligo treatment (Figure 6(b), *P* < 0.01).

## 4. Discussion

Resistin has been recognised as an adipocytokine positively correlated with insulin resistance and type 2 diabetes and is predictive of cardiovascular disease [15, 16]. However, the role of resistin in obesity associated with fatty liver disease remains controversial [17–19]. In the present study, we aimed to (1) identify whether the development of NAFLD induced by HFD feeding in mice is associated with overexpression of resistin mRNA and protein in adipose tissue and the liver; (2) observe whether inhibition of resistin expression with synthetic RNA oligo attenuates metabolic and histological abnormalities in NAFLD; and (3) clarify whether and which inflammatory cytokines and signalling molecules are involved in resistin-mediated hepatic insulin resistance and NAFLD.

This study discovered that, after a total of 14 weeks of HFD exposure, C57BL/6 mice developed abdominal obesity associated with hyperlipidaemia, hyperinsulinemia, and impaired GTT. Resistin mRNA and protein expressions were markedly increased in white adipose tissue of HFD control mice, which led to elevated circulating resistin levels. The previous study showed that elevated plasma resistin is associated with hepatic insulin resistance induced by 3 weeks' HFD feeding in mice and that treatment with antisense resistin suppressed hepatic glucose production [23]. However, that overexpression of resistin links to NAFLD has not been examined [23]. Our study showed that, on HFD exposure for 14 weeks, C57BL/6 mice developed moderate hepatic steatosis with elevated serum AST and ALT, indicating pathological changes of NAFLD in HFD mice. Interestingly, resistin mRNA and protein expressions were also significantly higher in liver tissue of HFD mice, suggesting that resistin is involved in HFD-induced NAFLD. Because resistin in rodents is primarily expressed by adipocytes the induced expression of resistin protein and mRNA in the liver is possibly secondary to hepatic fat accumulation in HFD mice. Furthermore, our study found that reduction of resistin with RNA oligo treatment ameliorated hyperinsulinemia and improved impaired GTT. Antiresistin oligo treatment also improved hyperlipidaemia and hepatic TG accumulation and normalized hepatic enzymes and the histological spectrum of hepatic steatosis in HFD mice. The NEFA levels in antiresistin treated mice were significantly reduced, consequently diminishing the influx of free FFAs to the liver, and thus attenuated FFAs-induced lipotoxicity. FFAs have direct hepatotoxicity through the induction of an endoplasmic reticulum stress response and subsequent activation of the mitochondrial pathway of cell death [26]. Lipotoxicity is a key pathogenic process in NAFLD, and it correlates with progressive inflammation and fibrosis [27].

Several lines of studies have shown a very strong and consistent association between resistin and inflammatory diseases in humans [8, 24, 25]. The mechanisms of NAFLD are closely linked to adipose dysfunction which increases secretion of proinflammatory cytokines, including TNF-*α*, IL-6, transforming growth factor-*β*, and retinol binding protein-4 [8, 22, 27–29]. TNF-*α* has been implicated in insulin resistance and liver fibrosis as well as advanced stages of NAFLD in humans [28]. IL-6, as a proinflammatory cytokine, has been proposed as a potential mediator leading to NAFLD [29]. In this study, we observed that elevated circulating resistin was associated with increased serum TNF-*α*, IL-6, and leptin; on the other hand, adiponectin was decreased in HFD fed mice. The elevated levels of serum resistin, TNF-*α*, IL-6, and hepatic enzymes suggest that resistin may contribute to NAFLD, at least in part, through upregulating systemic and hepatic inflammation.

The key finding of this study is that treatment with synthetic RNA oligo significantly reduced adipose mass and attenuated overexpression of resistin along with normalized serum TNF-*α* and IL-6 and enhanced adiponectin. Consequently, glucose intolerance and histopathological changes in the liver were improved by antiresistin RNA oligo treatment. Thus, it is highly likely that the events in the liver are due to the primary effects of the intervention on the adipose tissue events. A previous study showed that loss of resistin prevented hepatic steatosis is related to leptin deficiency [30]. In our study, reduced resistin expression and secession by RNA oligo neither significantly affected serum leptin levels nor altered leptin mRNA expression in adipose tissue. This study provided evidence to support the notion that resistin plays an important role in the pathogenesis of NAFLD but its effect is independent of the leptin pathway. Lowering resistin by RNA oligo with reduction of circulating TNF-*α* and IL-6 might provide a new insight into managing inflammation-mediated diseases, such as NAFLD, type 2 diabetes, and cardiovascular disease.

Our previous study showed that HFD-induced NAFLD in rats is associated with decreased insulin activation of glycogen synthase and increased gluconeogenesis [31], which, in turn, exacerbates hyperglycemia. In this study, antiresistin oligo treatment attenuated the elevated serum glucose, possibly through regulating hepatic gluconeogenesis and decreasing glucose production. Liver gluconeogenesis is driven by the availability of gluconeogenic substrates and the activity of PEPCK and glucose 6-phosphatase [32]. To understand whether antiresistin oligo also influences enzymes involved in gluconeogenesis, the expression of hepatic PEPCK was detected by qRT-PCR and Western blotting in liver tissues. We observed that the elevated serum level of resistin was associated with the expression of PEPCK in the liver of HFD mice. Furthermore, we found that inhibition of resistin with RNA oligo decreased PEPCK expression. Antiresistin RNA oligo treatment significantly reduced serum glucose level associated with downregulation of PEPECK expression, suggesting that inhibition of resistin may be a potential therapy for hyperglycemia through downregulation of hepatic PEPCK.

The hallmark of NAFLD is the presence of ectopic fat in the hepatocytes. The underlying mechanism of this pathological fat deposition, mainly triglycerides [33], is involved in an imbalance between lipid availability (from circulating lipid uptake or* de novo* lipogenesis) and lipid disposal (via FFA oxidation or triglyceride-rich lipoprotein secretion) which eventually triggers lipid peroxidative stress and hepatic injury [34]. In this study, HFD feeding caused hyperlipidemia associated with markedly elevated levels of serum AST and ALT, indicating hepatocytes injury and hepatic overproduction of lipids. The rate of hepatic lipogenesis is controlled by sterol regulatory element-binding proteins (SREBPs) which enhance transcription of genes encoding enzymes of triglycerides and fatty acid biosynthesis and uptake [35]. SREBPs have three subtypes, SREBP-1a, SREBP-1c, and SREBP-2. Among them, SREBP-1c is most highly expressed in brown fat, followed by the liver [36]. Previous study showed that SREBP-1c as a key transcription factor regulating the gene expression of key enzymes implicated in lipogenesis in the liver is experimentally connected to NAFLD [37]. To understand the mechanism underlying reduced hepatic lipid accumulation by antiresistin RNA oligo we measured SREBP-1c mRNA and protein expression in liver tissues of HFD mice with or without antiresistin RNA oligo treatment. We showed that elevated serum resistin was positively related to SREBP-1c mRNA and protein expression in the liver of HFD mice. Inhibition of resistin with RNA oligo was associated with markedly decreased SREBP-1c expression, which is likely to reduce hepatic triglyceride biosynthesis (*de novo* lipogenesis) and outflux. Conversely, previous study showed that increase in cellular neutral lipid content induced by human resistin is mediated through the induction of the cellular SREBP-1 and SREBP-2 lipogenic pathways [38]. A recent study reported that SREBP-1 expression and transcriptional activity were not affected in antiresistin siRNA 3T3-L1 cells [39]. The different findings between the current* in vivo* study and previous* in vitro* study by Ikeda et al. may be due to SREBP-1c gene selectively expressed in the liver and relevantly low level of SREBP-1 expression in 3T3-L1 cells. Together with increased lipid content associated overexpression of resistin and SREBP-1c in HFD mice, this study demonstrated that resistin promotes hepatic lipogenesis and hepatic steatosis through, at least in part, upregulating SREBP-1c. Inhibition of resistin may indirectly control hepatic lipogenesis pathway.

## 5. Conclusions

This study demonstrated that (1) downregulation of resistin expression in adipose tissue and the liver with decreased circulating resistin levels normalized HFD-induced hyperlipidaemia and hyperglycemia; (2) reduction of elevated serum resistin levels by antiresistin RNA oligo was associated with a reduction of circulating TNF-*α* and IL-6 but with no influence on serum leptin, indicating that resistin-mediated hepatic steatosis occurs through upregulation of inflammation independent of the leptin pathway; (3) antiresistin oligo may be capable of regulating gluconeogenesis and lipogenesis in the liver through downregulation of hepatic PEPCK and SREBP-1c expression. The effect of antiresistin RNA oligo in mice may not be directly translated to clinical study because the murine resistin protein has only 59% of similarity to human one [40, 41]. Our findings that inhibition of resistin with antisense RNA oligo ameliorated metabolic and histological abnormalities in animal model of NAFLD highlighted the role of resistin in pathogenesis of NAFLD. This study also demonstrated a correlative relationship between resistin and TNF-*α* as well as IL-6, suggesting the proinflammatory cytokines as the molecular link between murine resistin- and human resistin-mediated NAFLD.

## Figures and Tables

**Table 1:** The primer sequences.

Gene	Nucleotide	Sequence (5′ to 3′)	Product size (bp)	Accession number
*Retn *(resistin)	Sense	AGA CTG CTG GCC TTC TGG GC	194	AF323080.1
	Antisense	TTT CCT TTT CTT CCT TG		

*Tnf *(TNF-*α*)	Sense	ATGGCCTCCCTCTCATCAGT	97	NM_013693.3
	Antisense	TTTGCTACGACGTGGGCTAC		

*Il6 *(IL-6)	Sense	GAT GCT ACC AAA CTG GAT ATA ATC	247	NM_031168.1
	Antisense	GGT CCT TAG CCA CTC CTT CTG TG		

*Pck1 *(PEPCK)	Sense	TGCGGATCATGACTCGGATG	126	NM_011044
	Antisense	AGGCCCAGTTGTTGACCAAA		

*Scebp1 *(SREBP-1c)	Sense	CGGCTCTGGAACAGACACTG	185	NM_011480.3
	Antisense	CTCAGGAGAGTTGGCACCTG		

*Adipoq *(adiponectin)	Sense	GGAACTTGTGCAGGTTGGATG	171	NM_009605.4
	Antisense	CCCTTCAGCTCCTGTCATTCC		

*Lep *(leptin)	Sense	CACCAGGATCAATGACATTTCACA	71	NM_008493.3
	Antisense	TGAAGTCCAAGCCAGTGACC		

*Actb *(*β*-actin)	Sense	GTA CCA CTG GCA TCG TGA TGG ACT	323	NM_007393
	Antisense	CCG CTC ATT GCC AAT GGT GAT		

**Table 2:** Effects of antiresistin RNA oligo on body weight, metabolic parameters, and hepatic enzymes.

Paradigm parameter	Chow	HF-Con-oligo	HF-Res-oligo
After treatment Bwt (g)	28.5 ± 0.9	32.8 ± 1.2^a^	32.1 ± 1.7^d^
Epididymal fat (g/100 g Bwt)	1.58 ± 0.05	3.3 ± 0.12^b^	2.62 ± 0.32^ad^
Serum glucose (mM)	6.70 ± 0.75	9.59 ± 0.72^a^	7.22 ± 0.30^d^
Serum insulin (pg/mL)	528 ± 150	689 ± 121^a^	503 ± 164^d^
HOMA-IR	22.8 ± 1.9	50.1 ± 6.4^b^	27.5 ± 6.2^d^
Serum cholesterol (mM)	3.90 ± 0.09	6.87 ± 0.21^b^	4.95 ± 0.37^d^
Serum triglyceride (mM)	0.63 ± 0.01	0.98 ± 0.02^b^	0.79 ± 0.04^d^
Serum NEFA (mM)	0.78 ± 0.16	1.03 ± 0.08^a^	0.83 ± 0.03^d^
Serum ALT (U/L)	29.9 ± 0.6	89.6 ± 9.5^b^	52.1 ± 10.3^d^
Serum AST (U/L)	32.4 ± 7.9	199.8 ± 21.7^c^	65.8 ± 11.7^f^
Serum resistin (pg/mL)	513 ± 36	668 ± 35^b^	472 ± 19^e^

Mice were fed standard chow and high-fat diet for 10 weeks and then with 4-week i.p injection (3 times/week) of saline (Chow, 5 mL/kg of body weight), scrambled control oligo (HF-Con-oligo 25 mg/kg of body weight), or antiresistin RNA oligo (HF-Res-oligo, 25 mg/kg of body weight). Metabolic parameters are represented as mean ± SEM (*n* = 10 each group) after 4 weeks' treatment. Bwt: body weight; HOMA-IR: homeostatic model assessment-insulin resistance; TG: triglycerides; NEFAs: nonesterified fatty acids; AST: aspartate aminotransferase; ALT: alanine aminotransferase. ^a^ *P* < 0.05, ^b^ *P* < 0.01, and ^c^ *P* < 0.001 HF-Con-oligo group versus chow fed mice; ^d^ *P* < 0.05, ^e^ *P* < 0.01, and ^f^ *P* < 0.001 versus HF-Con-oligo mice.

## Abbreviations

ALT: Alanine aminotransferase
AST: Aspartate aminotransferase
AUC: Areas under the curve
HFD: High-fat diet
ILs: Interleukins
IPGTT: Intraperitoneal glucose tolerance test
Mets: Metabolic syndrome
NAFLD: Nonalcoholic fatty liver disease
NEFA: Nonesterified fatty acids
PEPCK: Phosphoenolpyruvate carboxykinase
RNA oligo: RNA oligonucleotide
SREBP-1c: Sterol regulatory element-binding protein-1c
TG: Triglycerides
TNF-*α*: Tumor necrosis factor-*α*.
